# Fatal Curiosity: A Case of Suicidal Attempt by Abrus Seeds Consumption Through Online Research

**DOI:** 10.7759/cureus.38458

**Published:** 2023-05-02

**Authors:** Mukesh Kumar, Prateek Kumar Dinkar, Haider Abbas, Esha Chaudhary

**Affiliations:** 1 Emergency Medicine, King George's Medical University, Lucknow, IND; 2 General Medicine, King George's Medical University, Lucknow, IND; 3 Internal Medicine, Government Medical College, Kannauj, IND

**Keywords:** medical attention, suicide, procurement, toxicity, abrus precatorius

## Abstract

*Abrus precatorius *(Jequirity, Indian liquorice, rosary bead, Gunja, or rati)* *seeds are highly toxic and are often ingested as a means of suicide in India. Gastric symptoms like bleeding, diarrhea, vomiting, and epigastric pain are the common manifestations of this toxicity. Abrin, a toxic substance found in the seeds, is structurally and functionally similar to ricin and is considered even more fatal. We report the first case of *Abrus precatorius* poisoning, where the internet was utilized to procure a potentially deadly poison with the intention to commit suicide in north India. Such actions are relevant to the medical field, particularly regarding the potential risks associated with the unsupervised procurement and misuse of toxic substances. The case highlights the potentially fatal consequences of ingesting Abrus seeds and the need for prompt medical attention in such cases.

## Introduction

*Abrus precatorius* is one of the deadliest poisons in the world, known commonly as rosary peas, jequirity bean, and crab's eye. Abrin, a toxic substance found in the seeds of *Abrus precatorius*, is structurally and functionally similar to ricin, a protein toxin found in castor beans (Ricinus communis). However, abrin is considered even more fatal than ricin [1, 2]. The ingestion of seeds among children is often seen unintentionally, as they are drawn to the seeds' vibrant colors. However, intentional ingestion of the seeds can also occur, especially among individuals aware of the potentially toxic nature of the seeds and seeking to end their own lives. The primary mechanism of abrin toxicity involves the inhibition of ribosomal protein synthesis, causing cellular death. An estimated human fatal dose of crushed seeds of Abrus is 0.1-1 μg/kg and has caused death after ingesting 1-2 crushed seeds [1,2]. Abrus seeds are considered non-poisonous if ingested whole due to the resistance of the harder outer shell against digestion. However, if chewed, the toxin is released, and the polypeptide B chain binds to the receptors on the intestinal cell surface while the polypeptide A chain binds to the cytoplasm. Upon entry into the cell, the A chain peptide acts on the 60 S ribosomal subunit, preventing the binding of elongation factor 2, thereby leading to the inhibition of protein synthesis and subsequent cell death [3,4]. 

## Case presentation

A 43-year-old female presented to our ED owing to the intentional ingestion of 25 crushed seeds of Abrus precatorius on July 24, 2022. Furthermore, the patient admitted to having the intention to commit suicide and surfed on the internet for the fatal dose and the effective ways of consumption of toxins available. On admission, she had complaints of abdominal pain, which was colicky in nature. After consumption of seeds, she developed multiple episodes of vomiting, which was orange in color initially and later changed to white color and associated with loose, watery stools. On physical examination, she looked unwell, with a temperature of 99.4°F, heart rate of 100 beats per minute, respiratory beat of 30 breaths per minute, blood pressure of 99/75 mm Hg, and oxygen saturation of 90% on room air. Her whole abdomen had diffuse mild tenderness without abdominal muscle tension and rebound tenderness. A 12 lead ECG showed sinus tachycardia without ectopy or arrhythmias. On respiratory examination, bilateral fine crepitations were audible over the chest. On day 1 of admission, her biochemical parameters were slightly deranged. Liver enzymes and bilirubin were normal except for serum alkaline phosphatase, which was 258.9 IU/L (50-240 IU/L). The renal function test was deranged with serum urea of 46.3 mg/dl (10-45 mg/dl) and serum creatinine of 2.21 mg/dl. Prothrombin time was >70 seconds (11-15 seconds), Troponin-T was 0.084 ng/ml (control of 0.014), and pro-B-type natriuretic peptide (pro-BNP) was 251.2 pg/ml (0-125) on day 1. On day 2, a psychiatrist on call diagnosed adjustment disorder (Δ F43.2). On day 1, she was admitted to the ICU to manage fluid and electrolyte status due to concern for delayed toxicity. On day 2, she continued vomiting and developed stools with blood, multiple episodes per day, associated with epigastric pain and tenderness in the abdomen. On day 3, the patient continued having diarrhea but stopped vomiting. On day 4, her sinus tachycardia and abdominal tenderness also resolved. There was no fever, headache, seizures, or ear discharge. Her vitals were stable, and she was maintaining adequate hydration. The central nervous system examination revealed a grade 1 sensorium with a Glasgow Coma Score (GCS) of 15. Both pupils were reactive to light. There was no papilledema. She was moving her limb on command, and her deep tendon reflex was intact with a bilateral plantar response. Detailed motor and sensory system examinations were carried out and found to be intact. There was no neck stiffness. We closely kept monitoring related parameters until the patient fully recovered. Treatment consisted of escitalopram, pantoprazole, ondansetron, tranexamic acid, and ceftriaxone 1 gm IV twice daily was added empirically. Her sensorium remained adequate, with satisfactory bowel and bladder movement. On day 3, liver enzymes and bilirubin were normal except serum protein 5.45 gm/dl (6.0-7.8) and serum albumin 3.34 gm/dl (3.5-5.0 gm/dl). On day 5, she maintained stable hemodynamic parameters and shifted into the ward from ICU. On day 7, liver enzymes improved and became normal. Prothrombin time was decreased to 20.9 seconds (11-15 seconds) on day 7. She was discharged after an eight-day hospital stay for outpatient follow-up in the department of psychiatry without any sequela.

## Discussion

**Figure 1:**
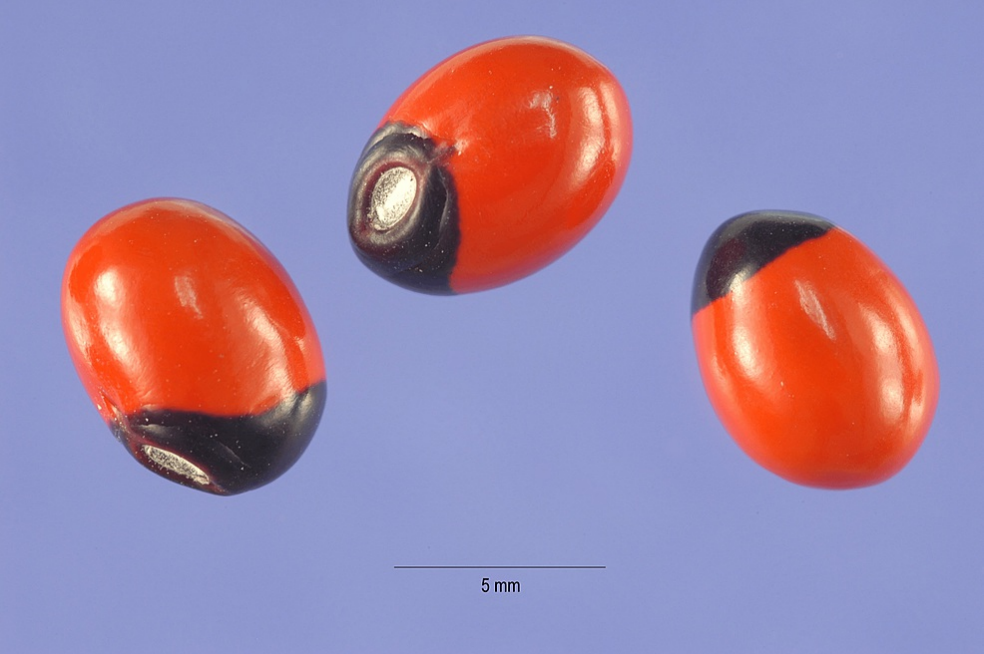
Colorful seeds of Abrus precatorius. Source: Wikimedia [11]

The estimated fatal dose of abrin in humans after oral consumption ranges between 0.1 and 1.0 µg/kg, which is equivalent to 1-3 seeds [5]. The seeds of *Abrus precatorius *contain abrin in a concentration of approximately 0.15% w/w (Figure 1). The patient attempted suicide by deliberately consuming *Abrus precatorius* crushed seeds. The decision to do so was made following online research on effective methods and fatal dosages. The patient was managed conservatively since she did not present to the hospital until 24 hours after ingestion. At that point, management options like gastric lavage or activated charcoal would yield no benefit. When the entire seeds are ingested, there are usually no detectable clinical symptoms, as the sturdy outer coating of the seeds allows them to travel through the GI tract without causing any harm. However, when the seeds are chewed, abrin, a toxic substance, is released. The GI tract poorly absorbs abrin, causing vomiting and diarrhea. This can lead to hypovolemia and imbalances in electrolytes, which can be serious and life-threatening, especially in regions with less developed healthcare systems. There has not been any published literature that could establish a significant relationship between prothrombin time (PT) and Abrus poisoning. As we observed that the PT was more than 70 seconds on the day of admission, it decreased to 20.9 seconds one day before she was discharged. On day 2, a psychiatrist on call diagnosed adjustment disorder (Δ F43.2). The patient fully recovered over an eight-day hospital stay even though she ingested a potentially fatal dose of Abrus [5,6]. The case reports published between 2010 and 2023 indicated that Abrus poisoning could result in various causes of death, including hypovolemia, multi-organ failure, diarrhea, shock, pulmonary edema, raised intracranial tension (ICT), and renal failure [1,7,8,9]. Only two case reports of Abrus poisoning have been published between 2005 and 2023, and both have reported fatalities. [9, 10]. In our study, the consumption of seeds was seven times the fatal dose estimated, but the patient recovered in an eight-day hospital stay. 

## Conclusions

The correlation between Troponin T, Pro-BNP, and Abrus poisoning needs to be established. This could be evident in ruling out cardiac dysfunction, if any, due to abrin toxin. We reported that the patient recovered quickly and was discharged with a diagnosis of adjustment disorder. Abrus seeds poisoning is a serious issue that can be fatal. It is crucial to understand the dangers associated with the consumption of these seeds and to avoid using them for any purpose, including suicide. While this is the third case of Abrus poisoning reported over the past 18 years, it is essential to note that any instance of poisoning, intentional or accidental, is concerning and requires prompt medical attention. It is crucial to take necessary precautions and avoid using substances that can be harmful to one's health. Addressing mental health concerns and seeking professional help when needed is important. Amid the escalating prevalence of mental health issues globally, individuals may resort to online resources to identify potentially lethal toxic substances. Thus, having a documented case of this nature could serve as a valuable reference point for healthcare professionals who encounter patients presenting with comparable symptoms.
